# Validation of the Nociception Level Index for the Detection of Nociception and Pain in Critically Ill Adults: Protocol for an Observational Study

**DOI:** 10.2196/60672

**Published:** 2025-02-28

**Authors:** Céline Gélinas, Shiva Shahiri T, Han Ting Wang, Maria Cecilia Gallani, Walid Oulehri, Denny Laporta, Philippe Richebé

**Affiliations:** 1 Ingram School of Nursing Faculty of Medicine and Health Sciences McGill University Montreal, QC Canada; 2 Centre for Nursing Research and Lady Davis Institute Jewish General Hospital Montreal, QC Canada; 3 Division of Intensive Care Department of Medicine CHUM - Hospital Centre of University of Montreal Montreal, QC Canada; 4 Department of Medicine Faculty of Medicine University of Montreal Montreal, QC Canada; 5 Faculty of Nursing Laval University Quebec City, QC Canada; 6 Research Centre Quebec Heart and Lung Institute - Laval University Quebec City, QC Canada; 7 Division of Anesthesia, Resuscitation and Perioperative Medicine Strasbourg University Hospitals Strasbourg France; 8 Federation of Translational Medicine Faculty of Medicine University of Strasbourg Strasbourg France; 9 Division of Adult Critical Care Jewish General Hospital Montreal, QC Canada; 10 Department of Medicine, Respiratory Division Faculty of Medicine and Health Sciences McGill University Montreal, QC Canada; 11 Department of Anesthesia and Resuscitation Polyclinic Bordeaux Nord Aquitaine Bordeaux France; 12 Department of Anesthesia and Pain Medicine Faculty of Medicine University of Montreal Montreal, QC Canada

**Keywords:** validation, NOL, Nociception Level, nociception, pain, intensive care unit, ICU, critical care, protocol

## Abstract

**Background:**

In the intensive care unit (ICU), many patients are unable to communicate their pain through self-reporting or behaviors due to their critical care condition, mechanical ventilation, and medication (eg, heavily sedated or chemically paralyzed). Therefore, alternative pain assessment methods are urgently needed for this vulnerable patient population. The Nociception Level (NOL) index is a multiparameter technology initially developed for the monitoring of nociception and related pain in anesthetized patients, and its use in the ICU is new.

**Objective:**

This study aims to validate the NOL for the assessment of nociception and related pain in critically ill adults in the ICU. Specific objectives are to examine the ability of the NOL to: (1) detect pain using standard criteria (ie, self-report and behavioral measures), (2) discriminate between nociceptive and nonnociceptive procedures, and (3) generate consistent values when patients are at rest.

**Methods:**

The NOL will be monitored in three ICU patient groups: (1) Group A, participants able to self-report their pain (the reference standard criterion using the 0-10 Faces Pain Thermometer) and express behaviors; (2) Group B, participants unable to self-report but able to express behaviors (the alternative standard criterion using the Critical-Care Pain Observation Tool); and (3) Group C, participants unable to self-report and express behaviors. The NOL will be tested before, during, and after two types of standard care procedures: (1) nonnociceptive (eg, cuff inflation to measure blood pressure, soft touch) and (2) nociceptive (eg, tube or drain removal, endotracheal or tracheal suctioning). Receiver operating characteristic curve analysis of the NOL will be performed for Groups A and B using pain standard measures as reference criteria. Mixed linear models for repeated measures will be used to compare time points, procedures, and their interaction in each group (A, B, and C). Based on power analyses and considering an attrition rate of 25%, a total sample size of 146 patients (68 in Group A, 62 in Group B, and 16 in Group C) is targeted.

**Results:**

This study was funded in April 2020 but could not be launched until 2022 due to the COVID-19 pandemic. Recruitment and data collection began at the primary site in July 2022 and has been implemented at the secondary sites in 2023 and 2024 and is planned to continue until 2026.

**Conclusions:**

The primary strength of this study protocol is that it is based on rigorous validation strategies with the use of pain standard criteria (ie, self-report and behavioral measures). If found to be valid, the NOL could be used as an alternative physiologic measure of pain in critically ill adults for whom no other pain assessment methods are available.

**Trial Registration:**

ClinicalTrials.gov NCT05339737; https://clinicaltrials.gov/study/NCT05339737

**International Registered Report Identifier (IRRID):**

DERR1-10.2196/60672

## Introduction

### Background

Patients in the intensive care unit (ICU) are exposed to many noxious stimuli as part of standard care, which may involve nociception and pain. Although nociception and pain are interrelated, they are distinct concepts. Nociception involves the neural processes of encoding noxious stimuli that lead to autonomic (eg, increased vital signs) and behavioral responses, which may or may not imply the sensation of pain [1,2]. Pain is a personal and multidimensional phenomenon influenced by biological, psychological, and social factors [1-3]. Appropriate detection of nociception and pain is key to providing adequate analgesia during critical illness in order to reduce the risk of adverse outcomes such as prolonged mechanical ventilation, longer ICU stay, and chronic pain development [4,5].

Many ICU patients experience pain at rest, and this pain is significantly increased during nociceptive procedures as part of standard care (eg, drain or tube removal, endotracheal suctioning, line insertion, wound care, and turning) [6-9]. Behavioral responses such as grimacing and muscle rigidity are commonly observed in ICU patients during standard care procedures and have been associated with their self-reported pain [10]. While the patient’s self-report (the reference standard) is the preferred method of pain assessment, it is unsuitable for patients unable to rate their pain due to a combination of several factors affecting their capacity to communicate, such as mechanical ventilation (>33% of Canadian ICU patients) [11], sedation, or an altered level of consciousness. In such situations, behavioral assessment tools such as the Behavioral Pain Scale (BPS) [12] and the Critical-Care Pain Observation Tool (CPOT) [13] are alternative standard measures of pain in patients unable to self-report with minimal motor function to exhibit behavioral responses [14]. However, these behavioral assessment tools cannot be used in heavily sedated patients or those receiving neuromuscular blocking agents as they become unresponsive to stimulation [15].

Alternative methods for pain assessment are necessary when none of the pain standard criteria (ie, self-report or behavioral measures) can be used. Although individual vital signs (eg, heart rate, blood pressure) are easily accessible through continuous bedside monitoring, they are not valid for ICU pain assessment due to inconsistent findings across studies and clinically insignificant variation in their values (<20%) [14,16]. However, research in the field of pain and anesthesia has shown that the combination of physiologic parameters is superior to their individual values [17]. Inspired by initial data from healthy subjects exposed to tonic heat stimuli inducing different pain levels [18], the Nociception Level (NOL) index (Medasense Biometrics Ltd) is a multiparameter technology, which was further developed for nociception monitoring and related pain in anesthetized patients [19-21]. In our recent review [22] of 6 studies in anesthetized patients, the NOL index outperformed single physiologic parameters for the detection of nociception during standard care procedures and experimental stimuli [17,20,23-25].

Although the validity of the NOL is supported in anesthesia, its use in the ICU is new. To our knowledge, we are the first research team to have pilot-tested the use of the NOL for nociception and pain assessment in the ICU. We conducted 2 pilot studies in 15 mechanically ventilated [26] and 54 cardiac surgery ICU patients [27] able to self-report their pain. In both studies, discriminative validation of the NOL was supported with higher index values during nociceptive procedures (ie, chest tube removal, endotracheal suctioning) compared with rest and a nonnociceptive procedure (ie, noninvasive blood pressure [NIBP] using cuff inflation). The NOL values were positively associated with self-reported pain and CPOT scores during nociceptive procedures, providing initial evidence of criterion validation with pain standard criteria [26,27]. Also, a NOL cutoff >25 was found to adequately classify patients with moderate to severe self-reported pain intensity (>4/10) [27]. However, these pilot validation studies of the NOL were limited to 2 nociceptive procedures as part of standard care and were solely conducted in ICU patients who were able to self-report. Further validation of the NOL during various standard care procedures in critically ill adults with different levels of consciousness or sedation and in response to analgesic treatment is necessary to confirm its validity for the assessment of nociception and related pain in the ICU.

### Study Rationale, Goal, Objectives, and Research Hypotheses

An instrument can only be shown as valid for a specific purpose in a given population and context [28]. The NOL index was initially developed and validated for nociception monitoring and related pain in anesthetized patients [17,19-25]. Therefore, validating its use for nociception (primary purpose) and pain assessment (related purpose) in critically ill adults admitted to the ICU (different population and context of care) is necessary. Inspired by our pilot work, strategies for this larger validation study include: (1) criterion validation (ie, NOL’s ability to detect pain according to pain standard criteria) and (2) discriminative validation (ie, NOL’s ability to discriminate between nonnociceptive and nociceptive procedures) [28]. Considering that reliability is a necessary condition for validity [28], test-retest reliability of the NOL’s ability to generate consistent values in similar conditions (eg, at rest) will also be examined.

Research Question: Is the NOL a valid method for the assessment of nociception and pain in critically ill adults in the ICU context?

In order to answer this research question, specific validation objectives are to examine the NOL’s ability to:

1. Detect pain in ICU patients able or not to self-report using appropriate pain standard criteria (ie, criterion validation);
2. Discriminate between nonnociceptive and nociceptive procedures part of standard care as well as before and after the administration of a breakthrough opioid dose (ie, discriminative validation);
3. Generate consistent values in similar conditions (ie, pre- and post-nonnociceptive and nociceptive procedures) when ICU patients are at rest.

Our research hypotheses to be tested include:

H1. The NOL index adequately classifies ICU patients with pain based on either their self-reported pain intensity or CPOT scores.

H2. The NOL index produces higher values during nociceptive procedures compared to nonnociceptive procedures and lower values post- versus preopioid administration.

H3. The NOL index generates consistent values pre- and post-nonnociceptive and nociceptive procedures when ICU patients are at rest.

## Methods

### Study Design

A prospective observational design was selected as it is appropriate for validation purposes using ICU standard care procedures in critically ill adults. The Strengthening the Reporting of Observational Studies in Epidemiology (STROBE) Statement: Guidelines for Reporting Observational Studies is used for the description and reporting of the study [29].

### Settings

This study is conducted in the ICUs of 3 tertiary-level university-affiliated health centers in Montréal, Québec, Canada. These ICUs have a total capacity ranging from 16-59 beds, and each ICU admits, on average, 1000-2750 patients annually.

### Participants

A consecutive sampling method is used to approach all eligible patients or their representatives during the study period. This sampling method aims to capture a representative sample of ICU patients able or not to self-report. Eligible ICU patients (Textbox 1) are assigned to one of the following groups:

1. Group A can communicate their self-report of pain and can exhibit behaviors (conscious and alert with Glasgow Coma Scale [GCS] [30] score of 13 to 15 or Richmond Agitation Sedation Scale [RASS] [31] score of 0);
2. Group B cannot communicate their self-report but can exhibit behaviors (altered level of consciousness with GCS score of 6 to 12 with a score ≥4 on the motor subscale or RASS score of –1 to –3); and
3. Group C cannot communicate their self-report or exhibit behaviors (unconscious with GCS score of 3 to 5 with a score ≤3 on the motor subscale or RASS score –4 or –5 or receiving neuromuscular blocking agents).

Exclusion criteria were selected to control for potentially confounding variables that may affect the NOL signal or pain standard measures. Patients assigned to Group A who screen positive for delirium are excluded as this condition is likely to affect the reliability of their self-report of pain [32]. Patients with conditions that may seriously influence perfusion of the hands, heart rate, heart rate variability, blood pressure, repetitive or high movements due to agitation or behavioral responses (eg, agitation, cognitive, or psychiatric conditions) are also excluded, thereby strengthening the internal validity of the study. Finally, patients with *Clostridium difficile* must be excluded because the required disinfection product (ie, sodium hypochlorite 5000-6000 ppm) could damage the NOL device, according to the manufacturer.

**Eligibility criteria of intensive care unit (ICU) patients.**

**Inclusion criteria:**

- Admitted to the ICU >24 hours
- Aged >18 years
- English or French speaking

**Exclusion criteria:**

- Delirium (Group A only)
- Lack an available finger
- Severe peripheral vascular disease affecting the upper limbs
- Uncontrolled cardiac arrhythmia (eg, atrial fibrillation)
- Pacemaker with paced rhythm
- Severe edema in upper limbs
- Hypoperfusion state or shock and receiving norepinephrine (>14 mcg/min) or equivalent vasopressors to maintain a systolic blood pressure >90 mmHg or a mean arterial pressure >65 mmHg
- Agitated (Richmond Agitation Sedation Scale +1 to +4)
- Psychosis
- Cognitive deficits (eg, Alzheimer)
- Positive for Clostridium difficile
- Pregnancy

### Recruitment and Consent Procedures

ICU patients are screened for eligibility by the research staff in collaboration with the medical and nursing team. Potentially eligible ICU patients able to self-report and consent, if they agree to participate, are assigned to Group A. Other potentially eligible ICU patients unable to self-report and consent are assigned to either Group B or C. According to Article 21 of the Quebec Civil Code for minimal risk studies, a significant person qualified to consent to care required by the state of health of the person of full age is asked to provide consent on their behalf for the research study.

Group A: Potentially eligible ICU patients able to self-report are approached for participation by their responsible nurse or physician. If interested, the research staff provides information about the study to the patient and obtain written informed consent.

Group B and Group C: For potentially eligible ICU patients unable to self-report, the person qualified to consent on their behalf is approached by their responsible nurse or physician. If interested, the research staff provides information about the study to the significant person and obtain written informed consent. If a patient participant who was previously unable to consent regains the ability to consent at any time, the research staff provides them with the informed consent form, allowing them to decide on their continued participation in the study. If a patient participant decides to withdraw from the study and requests their research data be removed, all collected information will be deleted.

### Study Procedures

Before starting data collection, the group assignment of the participant is confirmed by the research staff. Then, the research staff sets up the NOL device at the bedside, places the finger probe on the participant’s finger, and proceeds with calibration (requires less than 5 minutes). The screen of the NOL device is faced away from the patient and family members present in the room to reduce potential bias. Research staff also installs a video camera on a tripod at the foot of the bed to capture the face and the upper body in order to allow for interrater reliability examination of CPOT scores with another trained research staff not present at the bedside.

Participants are assessed at rest before, during, and 15 minutes after nonnociceptive and nociceptive procedures that are part of ICU routine care. Whenever possible, 15 minutes postprocedure are respected to allow for the resolution of the release, reaction, and elimination of stress hormones (ie, epinephrine and norepinephrine) and stress-activated responses that may be present following noxious stimulation [33]. The nonnociceptive procedures are ideally performed prior to nociceptive procedures and include soft touch (ie, research staff touching the patient’s arm for 1 minute) and cuff inflation for blood pressure measurement (NIBP), which were empirically shown to be painless in previous studies [34], including our pilot NOL studies [26,27]. In the sequence of nonnociceptive procedures, soft touch is performed first and cuff inflation last. Then, the goal is to observe up to two nociceptive procedures per participant (Textbox 2). Nociceptive procedures are selected according to the standard care required by the patient’s condition. These procedures do not involve mobilizing the patient out of bed, as important movements may introduce artifacts in the NOL data. When possible and as required by the patient’s condition, participants are also assessed before and 15 minutes postadministration of a breakthrough dose of an opioid such as fentanyl, morphine, or hydromorphone through a parenteral route (mainly through intravenous or subcutaneous routes) to capture onset or peak of action. The decision to administer an opioid is made as per local practice and based on an assessment performed by the ICU nurse or physician. Within a window of 48 hours after obtaining informed consent, a total of 10 to 12 time points of data collection is completed with each participant during their ICU stay (Table 1).

The NOL index is continuously monitored during the data collection time period, and values are extracted in a standardized manner as done in previous clinical studies for NOL data analysis purposes [24-27]. CPOT assessments (one-minute observation or duration of the procedure) are performed on participants from Groups A and B by research staff, and then conscious patients able to self-report (Group A) are asked to provide their self-report of pain intensity at each time point. Video recording is stopped before participants provide their self-report to avoid possible bias by the research staff, who will view the videos for CPOT scoring at a later time. Finally, demographic and clinical information is extracted from the participants’ medical files. At the end of data collection, the study equipment (ie, video camera, tripod, NOL device, and keyboard) is disinfected according to the prevention control infection procedures of each institution before being used for another patient.

**List of nociceptive and nonnociceptive standard care procedures in the intensive care unit.**

**Nociceptive procedures**

- Chest tube removal
- Drain removal
- Mouth, endotracheal, or tracheal suctioning
- Arterial line insertion
- Peripheral intravenous line insertion
- Subcutaneous injection
- Wound care
- Bed turning or repositioning

**Nonnociceptive procedures**

- Soft touch of the patient’s arm
- Noninvasive blood pressure cuff inflation (NIBP)

**Table 1:** Assessment time points and pain variables to be measured in each group (A, B, and C).

Measure	Nonnociceptive procedures					Nociceptive procedure 1				Nociceptive procedure 2				Analgesic^a^	
	Pre	Soft touch	Cuff inflation	Post	Pre		During	Post	Pre		During	Post	Pre		Post
NOL^b^	A^c^, B^d^, C^e^	A, B, C	A, B, C	A, B, C	A, B, C		A, B, C	A, B, C	A, B, C		A, B, C	A, B, C	A, B, C		A, B, C
Behaviors	A, B	A, B	A, B	A, B, C	A, B		A, B	A, B	A, B		A, B	A, B	A, B		A, B
Pain level	A	A	A	A	A		A	A	A		A	A	A		A

^a^Administration of an analgesic as required by the patient’s condition.

^b^NOL: Nociception Level Index.

^c^Group A: Patients are able to communicate their self-report of pain and can exhibit behaviors.

^d^Group B: Patients are unable to communicate their self-report of pain but can exhibit behaviors.

^e^Group C: Patients who cannot communicate their self-report of pain or exhibit behaviors.

### Variables and Measurement Tools

#### Primary Measure: The Nociception Level (NOL) Index–All Groups (A, B, and C)

**Figure 1:**
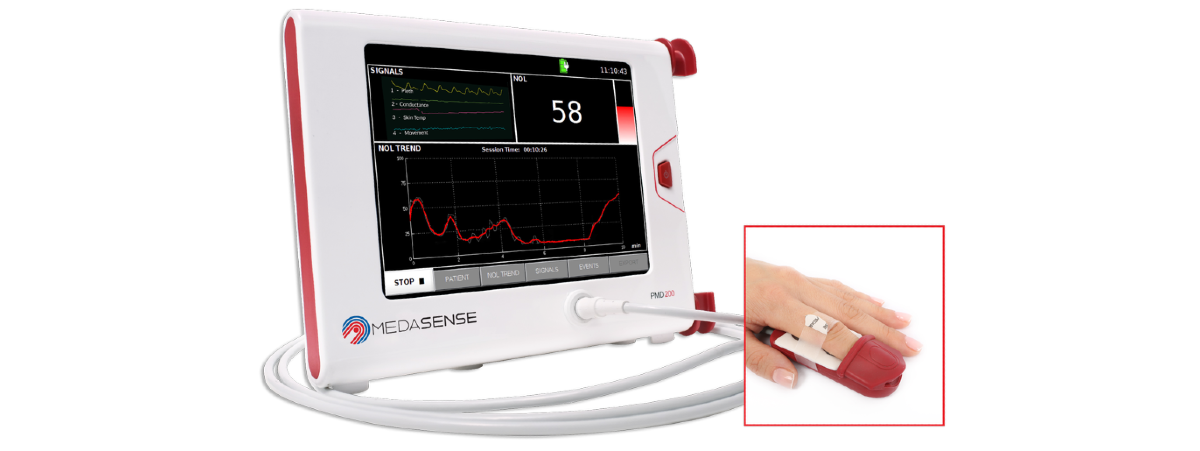
Nociception Level (NOL) index.

The Pain Monitoring Device-200 (PMD-200; Medasense Biometrics Ltd; Figure 1) is used in this study. PMD-200 offers the multiparametric NOL index (0-100) and was approved by Health Canada for clinical use in September 2017. The NOL captures several physiological parameters simultaneously through a finger probe and disposable sensor, which includes 4 small sensors, sampled 50-500 Hz: (1) accelerometer, (2) photoplethysmograph, (3) galvanic skin response, and (4) peripheral temperature. From these 4 sensors, the following physiological parameters are extracted: heart rate, heart rate variability, photoplethysmography pulse wave amplitude, skin conductance level, number of skin conductance fluctuations, skin temperature, and their time derivatives. All these parameters are integrated and analyzed simultaneously using a Random Forest machine learning model approach to provide the NOL index, which can range from 0 to 100 [17]. A NOL cutoff value >25 for the detection of self-reported pain was found in our pilot study with ICU cardiac surgery patients [27].

The PMD-200 device allows the documentation of each event (ie, beginning and end time of rest and procedure assessment) or any contextual information (eg, interruption during a procedure), which is facilitated with the use of a wireless keypad. All NOL data are electronically collected every 5 seconds by the PMD-200 monitor.

#### Reference Standard Measure of Pain–Group A

**Figure 2:**
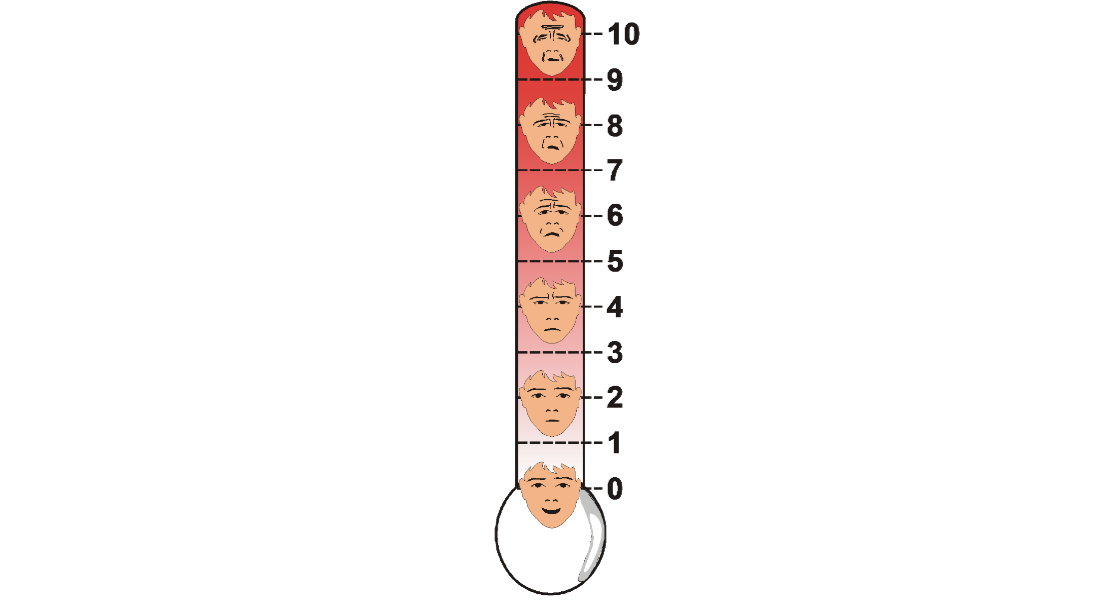
The 0-10 Faces Pain Thermometer (FPT)[35].

The Faces Pain Thermometer (FPT) was selected to measure pain intensity as it can accommodate adult patients of various ages. It consists of an enlarged visual thermometer including 6 faces with a numeric rating scale scoring from 0=“no pain” to 10=“worst possible pain” (Figure 2). The validity and reliability (test-retest) of the tool were established in ICU patients [35]. The FPT was used in many previous validation studies led by the primary investigator [13,34,36] and pilot NOL studies [26,27].

#### Alternative Reference Standard Measure of Pain–Groups A and B

The Critical-Care Pain Observation Tool (CPOT) [13] was selected for behavioral pain assessment in critically ill adults. The CPOT was used in our NOL pilot studies [26,27], and it is 1 of the 2 behavioral scales suggested as alternative reference standard measures of pain by the Society of Critical Care Medicine [14] and has been implemented in clinical practice in the 3 study ICUs. The CPOT includes 4 behavioral items: (1) facial expressions, (2) body movements, (3) muscle tension, and (4) compliance with the ventilator (for mechanically ventilated patients) or vocalization (for nonintubated patients). Each item is scored from 0-2, and the total score can range from 0-8. The CPOT has been validated in 47 studies with more than 3900 ICU patients with surgical, trauma, and medical diagnoses from various countries [34]. Consistent findings of good interrater reliability (interclass correlation coefficient [ICC] >0.60 between research staff and ICU nurses) and ability to discriminate between various nociceptive and nonnociceptive procedures were found across studies. Criterion validation of the CPOT was also supported by positive correlations (*r*>0.40) with ICU patients’ self-reported pain intensity [34]. A CPOT score >2 was found to adequately identify patients with self-reported pain (AUC>0.80) [34,36].

The research staff of all sites have been trained to use the CPOT using a standardized training session previously described [37]. Briefly, this 60-minute training session covers the description of the CPOT content and its scoring process. Patient videos are viewed to practice scoring with the tool with the goal of reaching consistent CPOT scores between raters within a total score difference of no more than 1 point.

#### Demographic and Clinical Information for ICU Patient Participants

Demographic information (eg, age, sex, gender, and ethnicity) is collected from the patient or their representative. Information related to the patient’s clinical condition (ie, ICU admission diagnosis, mechanical ventilation, the severity of illness [Acute Physiology and Chronic Health Evaluation; APACHE II] [38], level of consciousness [GCS] [30], sedation level [RASS] [31], delirium screening with the Confusion Assessment Method-ICU [39] or Intensive Care Delirium Screening Checklist [40], according to the respective tool used at each site), as well as medication information (ie, morphine equivalent doses [41] received within 4 hours before and during the entire data collection, sedative agents, vasopressors, and use of opioids before ICU admission) is extracted from the medical records by the research staff. The following clinical information is collected at the time of obtaining consent and performing data collection: mechanical ventilation status, GCS score, RASS score, and delirium screening.

### Sample Size Calculation

The primary objective is to validate the NOL using pain standard criteria (ie, self-report and CPOT scores). Therefore, ROC is considered our primary analysis and guides the sample size calculation for Group A and Group B. Power calculations were based on our pilot findings [26,27] and results from previous ICU studies considering a clinically acceptable AUC of 0.75 [42] (using a null hypothesis of AUC=0.50) with a power of 80% and an adjusted α of .025 (2 ROC curves for each procedure in each group). For Group A in whom the self-report of pain is obtained and using a ratio of 2:1 in pain:no pain cases according to self-reported pain intensity scores of <4 and >4, a sample of 54 patients is required. For Group B in whom only the CPOT scores are available and using a ratio of 1.5:1 in pain:no pain cases according to CPOT scores of <2 (no pain) and >2 (pain), a sample of 50 patients is necessary. For Group C (unable to self-report and express behaviors), we will not be able to use any pain standard criterion. Therefore, our main objective is to test whether the NOL can discriminate between a nociceptive procedure and a nonnociceptive procedure. In order to detect a minimal mean increase of 10 in the NOL value during the nociceptive procedure (with a standard deviation of 10 as found in our pilot findings), a minimal sample size of 13 patients is required to run paired *t* tests with a power of 80% and an α of .01 (to account for multiple test comparisons). Considering an attrition rate of 25% (data collection was completed in 73% of consenting patients in our pilot work), recruitment targets include 68 patients in Group A, 62 patients in Group B, and 16 in Group C for a total of 146 ICU patients. Power calculations were performed by a statistician using Power and Precision 4 (Biostat Inc).

### Data Analysis

SPSS software (version 29; IBM) and SAS (version 9.4; SAS Institute) will be used for data analysis. Descriptive statistics will be computed to characterize the study samples and outcomes. The NOL signal quality will be checked for all enrolled patients to ensure the accuracy of the data. As done in previous studies, NOL values will be averaged within 15 seconds before and after the peak value obtained after the start of each nonnociceptive procedure (7 NOL values total around the peak for soft touch and NIBP) and nociceptive procedure. In addition, NOL values will be averaged over a 1-minute period at rest before and after the procedure. CPOT scores will be obtained independently by 2 trained raters, one at the bedside and one who will view the patient videos at a later time. Intraclass correlation coefficients (ICC) will be calculated between the CPOT scores of both raters during procedures. ICC>0.80 will confirm excellent interrater reliability [28].

For objective 1 (criterion validation), the receiver operating characteristic (ROC) curve [43] will be performed to examine the ability of the NOL to adequately classify patients with pain. In patients able to self-report (Group A), an established pain intensity score >4 based on participants’ self-reports as the reference standard criterion indicating moderate to severe pain will be used [10,44]. This criterion is also commonly used in practice to support clinical decisions in the administration of opioids. A ROC curve will be obtained for each nociceptive procedure (a total of 2 procedures per patient). In patients unable to self-report but in whom behaviors can be observed (Group B), we will use the determined CPOT score >2 as the alternative reference criterion, which is associated with the presence of moderate to severe pain [44]. Again, for Group B, a ROC curve will be obtained for each nociceptive procedure (total of 2 procedures per patient) [45]. As a supplementary analysis, we will perform ROC curves for repeated-measures design in each group (A and B) and compare them [46]. In addition, considering that the CPOT will be available in Group A and Group B, ROC curves will be performed in each group separately to compare the AUC of independent curves for each of the 2 procedures [45]. The NOL cutoff value that will optimize both sensitivity and specificity will be determined.

Discriminative validation (objective 2) is our main objective to test in Group C, and paired *t* tests comparing the NOL values of each nociceptive procedure with the nonnociceptive procedure will be obtained. Generalized linear mixed model for repeated measures will allow us to compare the NOL index values across time points (pre, during, and post), procedures (nonnociceptive vs nociceptive), and their interaction in each group (A, B, and C). The generalized linear mixed model technique incorporates the full-information maximum likelihood procedure, which allows parameter estimation for missing data [47]. NOL values are expected to be significantly higher during nociceptive procedures in all 3 groups. Covariates (eg, sex, gender, morphine equivalent doses, opioid use before ICU admission) may be added if appropriate. Finally, NOL data obtained at pre- and postadministration of an opioid dose will be tested separately using paired *t* tests as not all patients will be candidates for these assessments.

For test-retest reliability (objective 3), paired *t* tests will be performed between pre and post-nonnociceptive and nociceptive procedures in all 3 groups. Nonsignificant paired *t* tests are expected to support the stability of NOL values when the patients are at rest.

### Ethical Considerations

Ethical approval was submitted in February 2022 and approved by the Medical and Biomedical Research Ethics Committee of the primary site in June 2022 (project # MP-05-2022-2988). A first amendment of the research protocol (version 2) was approved in June 2023 regarding the addition of pacemaker as an exclusion criterion as it affects the generation of the NOL signal. A second amendment of the research protocol (version 3) was approved in September 2024 for the addition of a third study site and revision of the sample size. According to our pilot studies [26,27], participating in this study is not associated with any known risks; however, participants will be informed that if they find the finger probe uncomfortable, they can choose to switch fingers or hands or have it removed to stop data collection. Participation in this research project is completely voluntary. Participants or their representatives have the freedom to decline or withdraw from the study at any time without providing a reason, with no consequences. Opting not to participate or withdrawing will not affect the quality of care and services they are entitled to receive. Participants or their representatives can also choose not to answer specific questions or decline video recording if they prefer.

The confidentiality of collected data, video recordings, and contact information will be maintained. Electronic folders will be saved on the secure server of the institution and their access will be restricted to only the research team members with their own username and password. Mechanisms to ensure confidentiality will include the assignment of numeric codes and the removal of personal identifiers. The code key linking the participants to their research file will be kept in a file protected by a unique password, stored separately from the coded research data, and only accessed by the research team. Informed consent forms will also be stored separately from the coded research data. The REDCap (Research Electronic Data Capture; Vanderbilt University) [48] web application licensed by the primary site will be used to create the data collection forms and to facilitate data storage and management between the study sites.

For monitoring control, safety and security, the research files may be examined by a person mandated by Canadian or international regulatory authorities, such as Health Canada, as well as authorized representatives of the study sponsor (the institution) or the research ethics board. The data will be permanently destroyed after 15 years. All participants have the right to access their study files at any time to verify and correct the information if necessary. They are informed and consented to the fact that summarized deidentified data will be presented in scientific conferences and publications.

## Results

This study was funded in April 2020 (see the funding report in Multimedia Appendix 1) but could not be launched until 2022 due to the COVID-19 pandemic. It was registered to ClinicalTrials.gov (NCT05339737) in April 2022. Recruitment and data collection began at the primary site in July 2022 and has been implemented at the secondary sites in 2023 and 2024. Recruitment and data collection will be ongoing until 2026.

## Discussion

In alignment with our pilot findings [26,27], we anticipate that the NOL will be able to detect pain according to pain standard criteria and to discriminate between nociceptive and nonnociceptive procedures. The enrollment of critically ill adults with various clinical conditions and the capacity to communicate will allow us to better generalize our validation findings to the ICU population.

### Methodological Strengths

The selection of rigorous validation strategies according to methodological guidelines in health measurement is the main strength of this study. With the participation of conscious ICU patients able to self-report, we can use the reference standard of pain, that is, self-reporting, for criterion validation. Furthermore, this validation strategy is reinforced by using the alternative reference standard, that is, the CPOT. Regarding discriminative validation, 2 strategies were selected, that is, discrimination between nonnociceptive and nociceptive procedures as well as before and after opioid administration. Test-retest reliability pre- and post-nonnociceptive and nociceptive procedures will allow us to examine the stability of the NOL values when patients are at rest. Both reliability and validation strategies are necessary to confirm the validity of an instrument [28].

In order to reduce potential bias related to pain scoring, Group A participants who are able to self-report will be blinded to the NOL values screen during the procedures. CPOT scoring will be completed by research staff before obtaining the patient’s self-report (ie, Group A participants) so raters are not influenced by self-reported pain intensity scores. Interrater reliability of CPOT scores will also be examined.

Our study lies in its foundation built upon the robust pilot work of the NOL conducted in the ICU setting. Our previous pilot studies have provided valuable insights and preliminary data to inform and support the research hypotheses and methodological decisions made in this study protocol. By expanding a rigorous validation process of the NOL to patients who are representative of the broader ICU population, the knowledge gained could contribute to identifying an alternative measure of pain in the most vulnerable critically ill adults.

### Potential Limitations and Mitigation Strategies

Recruiting ICU patients or obtaining written consent from the persons qualified to consent for them is challenging. This requires daily screening of eligible patients and close follow-up. Support from the nursing staff and physicians is key to identifying eligible patients and approaching those qualified to consent for them. Our research team has developed efficacious recruitment procedures, trained competent research staff, and established close collaboration with ICU care teams in all sites, which facilitate the implementation of this study. The feasibility of recruitment was also demonstrated in our pilot work. A screening log will be completed at each site to compile eligibility criteria and reasons for refusals, losses, or withdrawals. In our pilot studies [26,27], we were unsuccessful in obtaining data from some participants mainly due to missing the procedure, either because temporary fellows forgot to inform the research team or the procedure occurred after ICU discharge. Chest tube removal and endotracheal suctioning were the sole nociceptive procedures included in our pilot studies. Broadening the selection of nociceptive procedures may reduce the number of missed observations of these events. However, we may still miss some procedures due to urgent situations. Also, data collection will be planned as soon as possible after obtaining written consent to minimize losses. In addition, we will document any challenges related to the use of NOL (eg, absence or loss of signal) and troubleshooting strategies. Finally, CPOT raters cannot be blinded to the procedures they will view on the videos. As a result, they may anticipate patients’ behavioral responses to the procedure, which may influence their ratings. The examination of interrater reliability of CPOT scores involving trained research staff from different settings and using 2 methods of rating (bedside and video) may help minimize this potential bias.

### Conclusion

The validity of the NOL has been supported in anesthetized patients, but its use in the ICU context is still new. Its validation in the ICU is relevant as many critically ill adults may be unable to self-report or express pain behaviors. As part of the validation process, it is key to validate the NOL with patient groups in whom pain standard criteria can be used as well as during nociceptive and nonnociceptive procedures in order to support its validity for both nociception and related pain assessment in the ICU. If found to be valid, the NOL could be used as an alternative measure to detect nociception and pain and to guide pain management decisions in the most vulnerable, critically ill adults.

## Appendix

Multimedia Appendix 1

## Abbreviations

APACHE: Acute Physiology and Chronic Health Evaluation
BPS: Behavioral Pain Scale
CPOT: Critical-Care Pain Observation Tool
FPT: Faces Pain Thermometer
GCS: Glasgow Coma Scale
ICC: interclass correlation coefficient
ICU: intensive care unit
NIBP: noninvasive blood pressure
NOL: Nociception Level
NRS: Numeric Rating Scale
RASS: Richmond Agitation Sedation Scale
REDCap: Research Electronic Data Capture
ROC: receiver operating characteristic
STROBE: Strengthening the Reporting of Observational Studies in Epidemiology

## Data Availability

The dataset generated during and analyzed during this study is not publicly available due to ethical agreement but may be available from the corresponding author on reasonable request.
