# Supplementary material for: Validation of the Nociception Level Index for the Detection of Nociception and Pain in Critically Ill Adults: Protocol for an Observational Study
Source: JMIR Res Protoc. 2025 Feb 28;14:e60672. doi: 10.2196/60672 (PMC11909487; doi:10.2196/60672)
Supplement: Multimedia Appendix 1 [file resprot_v14i1e60672_app1.pdf]

**Review Type / Type d'évaluation:**	Reviewer 1 / Évaluateur 1
**Name of Applicant / Nom du chercheur:**	Gelinas, Céline
**Application No. / Numéro de demande:**	427389
**Agency / Agence:**	CIHR/IRSC
**Competition / Concours:**	Project Grant/Subvention Projet
**Committee / Comité:**	Psychosocial, Sociocultural & Behavioural Determinants of Health/Déterminants psychosociaux, socioculturels et comportementaux de la santé
**Title / Titre:**	Validation of a new multi-parameter technology to better diagnose pain in the adult intensive care population: The Nociception Level (NOL) Index

Adjudication Criteria/Critères de sélection

Significance and Impact of the Research/Importance et impact de la recherche: 4.5

Approaches and Methods/Approches et méthodes: 4.3

Expertise, Experience and Resources/Expertise, expérience et ressources: 4.4

Top/Bottom Selection/Groupe supérieur/inférieur

- (●) **Top/Groupe supérieur**
- ( ) **Bottom/Groupe inférieur**

**Review Type / Type d'évaluation:**	Reviewer 1 / Évaluateur 1
**Name of Applicant / Nom du chercheur:**	Gelinas, Céline
**Application No. / Numéro de demande:**	427389
**Agency / Agence:**	CIHR/IRSC
**Competition / Concours:**	Project Grant/Subvention Projet
**Committee / Comité:**	Psychosocial, Sociocultural & Behavioural Determinants of Health/Déterminants psychosociaux, socioculturels et comportementaux de la santé
**Title / Titre:**	Validation of a new multi-parameter technology to better diagnose pain in the adult intensive care population: The Nociception Level (NOL) Index

Summary of Application/Résumé de la demande:

This observational study will recruit a sample of 411 ICU patients, including one group of patients who is able to communicate (self-report) pain and who exhibit pain-related expressions/behaviors, a group who cannot communicate but can exhibit pain behaviors, and a third group who cannot communicate pain verbally nor express it behaviorally. All patients will be evaluated with the PMD, which yields a Nociception Level (NOL) index ranging from 0 to 100. This pain intensity index, which is derived from the recordings of several physiologic parameters (heart rate, heart rate variability, pulse wave amplitude, skin conductance, and peripheral temperature), will be compared to patient's subjective (Faces Pain Thermometer) and clinician/behavioral measures of pain (Critical Care Pain Observation – CPOT).

**Review Type / Type d'évaluation:**	Reviewer 1 / Évaluateur 1
**Name of Applicant / Nom du chercheur:**	Gelinas, Céline
**Application No. / Numéro de demande:**	427389
**Agency / Agence:**	CIHR/IRSC
**Competition / Concours:**	Project Grant/Subvention Projet
**Committee / Comité:**	Psychosocial, Sociocultural & Behavioural Determinants of Health/Déterminants psychosociaux, socioculturels et comportementaux de la santé
**Title / Titre:**	Validation of a new multi-parameter technology to better diagnose pain in the adult intensive care population: The Nociception Level (NOL) Index

Strengths and Weaknesses/Forces et faiblesses:

This is the third submission of an application that proposes to validate a new measure of pain, the Pain Measurement Device (PMD), to be used with patients in the Intensive Care Unit (ICU).

The revised application retains its original strengths including its focus on a highly relevant topic, a well-designed study using innovative technology to measure pain, excellent pilot data, and a well-qualified research team. In addition, the proposed study has the potential for producing a clinically useful assessment tool for measuring pain in patients who are unable to communicate their pain verbally or express it behaviorally.

There is also a solid scientific and clinical rationale supporting this application. Although patients' self-report is the gold standard for measuring pain, ICU patients are often unable to communicate due to their critical care condition requiring mechanical ventilation and administration of sedative agents which may alter levels of consciousness. Hence, the need to need to have access to a different technology to measure pain and improve its management.

Previously, there were concerns about the study feasibility, sample size calculation, statistical analyses, lack of consideration of sex and gender, and about knowledge translation strategies. These issues were addressed adequately in this revision. For instance, the sample size is now based on power analyses using more stringent criteria than before; in addition, two separate ROC curves taking into account differences between patients who can and those cannot communicate verbally their pain. Additional analyses are also planned to address potential sex and gender differences in pain ratings/intensity.

The issue of feasibility is addressed more thoroughly in this revised application and the projected pace of recruitment appears realistic based on the applicant's previous work with ICU patients. Nonetheless, the study protocol is rather complex and recruiting patients in ICU and obtaining written consent may still represent significant challenges in implementing the proposed study. Despite experience working with this population in the ICU environment and established collaborations with ICU staff, there is still some concern about feasibility and a plan b should be considered if recruitment does not flow as planned.

In addition to recruiting patients, the logistic of the study and the practical implementation of the assessment protocol are likely to be challenging as well. For instance, the assessment procedures involve obtaining multiple measures of pain under three different circumstances (nociceptive procedure, non-nociceptive procedures, and before and after opioid administration to control pain). There will be 9 to 11 assessments per patient (i.e., 3 procedures x 3 time points, and pre/post-opioid administration) with each participant during their ICU stay. The applicant provides convincing arguments and evidence that it can be done with her team and with the collaborating ICU staff.

The NOL index will be validated against patient's subjective (Faces Pain Thermometer) and clinician/behavioral measures of pain (Critical Care Pain Observation – CPOT), which are apparently the standards of references for measuring pain in

ICU patients. The application did not provide a critical discussion of other subjective and behavioral pain assessment tools and did not justify clearly, why these two were selected for the present study, except for the fact that the principal applicant has developed and used them previously.

Despite some limitations, this innovative technology could provide a valuable alternative measure of pain and improve its diagnosis and management non only in ICU patients but also in other vulnerable populations such as those in palliative care, older adults with severe cognitive deficits and infants and non-verbal children for which pain or nociception assessment is a major issue.

The principal applicant, Dr. Gélinas, is well qualified to conduct the proposed study. She is a mid-career, FRQS research career scientist, who completed a PhD in nursing and measurement in 2004 and postdoctoral training in nursing in 2006. She is Associate Professor at McGill University. She has a track record of successfully conducting validation studies of pain assessment tools and technologies in the ICU including in adults with impaired capacity. She has assembled a solid research team with experience using the different assessment tools. The co-principal applicant, Dr. Richebé, is a physician specialized in Anesthesiology and Intensive Care Medicine. In addition, he completed a PhD in neurosciences and neuropharmacology in 2005. He is Associate Professor and Director of Research for the Department of Anesthesiology and Pain Medicine at the Université de Montréal.

**Review Type / Type d'évaluation:**	Reviewer 1 / Évaluateur 1
**Name of Applicant / Nom du chercheur:**	Gelinas, Céline
**Application No. / Numéro de demande:**	427389
**Agency / Agence:**	CIHR/IRSC
**Competition / Concours:**	Project Grant/Subvention Projet
**Committee / Comité:**	Psychosocial, Sociocultural & Behavioural Determinants of Health/Déterminants psychosociaux, socioculturels et comportementaux de la santé
**Title / Titre:**	Validation of a new multi-parameter technology to better diagnose pain in the adult intensive care population: The Nociception Level (NOL) Index

Budget Recommendation/Recommandation budgétaire:

Budget is appropriate as requested.

**Review Type / Type d'évaluation:**	Reviewer 1 / Évaluateur 1
**Name of Applicant / Nom du chercheur:**	Gelinas, Céline
**Application No. / Numéro de demande:**	427389
**Agency / Agence:**	CIHR/IRSC
**Competition / Concours:**	Project Grant/Subvention Projet
**Committee / Comité:**	Psychosocial, Sociocultural & Behavioural Determinants of Health/Déterminants psychosociaux, socioculturels et comportementaux de la santé
**Title / Titre:**	Validation of a new multi-parameter technology to better diagnose pain in the adult intensive care population: The Nociception Level (NOL) Index

Please indicate your appraisal of the integration of sex as a biological variable as a strength, weakness, or not applicable to the proposal./Prière de sélectionner une option pour donner votre évaluation de l'intégration du sexe comme variable biologique en tant que point fort ou point faible de la proposition, ou en tant qu'élément non applicable à la proposition.

- ◉ **Strength/Point fort**
- ○ **Weakness/Point faible**
- ○ **Not applicable/Non applicable**

Please indicate your appraisal of the integration of gender as a socio-cultural determinant of health as a strength, weakness, or not applicable to the proposal./Prière de sélectionner une option pour donner votre évaluation de l'intégration du genre comme déterminant socioculturel de la santé en tant que point fort ou point faible de la proposition, ou en tant qu'élément non applicable à la proposition.

- ◉ **Strength/Point fort**
- ○ **Weakness/Point faible**
- ○ **Not applicable/Non applicable**

**Review Type / Type d'évaluation:**	Reviewer 1 / Évaluateur 1
**Name of Applicant / Nom du chercheur:**	Gelinas, Céline
**Application No. / Numéro de demande:**	427389
**Agency / Agence:**	CIHR/IRSC
**Competition / Concours:**	Project Grant/Subvention Projet
**Committee / Comité:**	Psychosocial, Sociocultural & Behavioural Determinants of Health/Déterminants psychosociaux, socioculturels et comportementaux de la santé
**Title / Titre:**	Validation of a new multi-parameter technology to better diagnose pain in the adult intensive care population: The Nociception Level (NOL) Index

Sex and/or Gender Considerations/Notions de sexe et/ou de genre:

The influence of sex and gender on the NOL will be examined with descriptive statistics stratified by sex and gender to characterize the study samples and outcomes. Sex and gender will also be used as covariates in the analyses. Interactions with groups we will be analyzed as well as associations with outcomes.

**Review Type / Type d'évaluation:**	Reviewer 2 / Évaluateur 2
**Name of Applicant / Nom du chercheur:**	Gelinas, Céline
**Application No. / Numéro de demande:**	427389
**Agency / Agence:**	CIHR/IRSC
**Competition / Concours:**	Project Grant/Subvention Projet
**Committee / Comité:**	Psychosocial, Sociocultural & Behavioural Determinants of Health/Déterminants psychosociaux, socioculturels et comportementaux de la santé
**Title / Titre:**	Validation of a new multi-parameter technology to better diagnose pain in the adult intensive care population: The Nociception Level (NOL) Index

Adjudication Criteria/Critères de sélection

Significance and Impact of the Research/Importance et impact de la recherche: 4.5

Approaches and Methods/Approches et méthodes: 4.5

Expertise, Experience and Resources/Expertise, expérience et ressources: 4.5

Top/Bottom Selection/Groupe supérieur/inférieur

- (●) **Top/Groupe supérieur**
- ( ) **Bottom/Groupe inférieur**

**Review Type / Type d'évaluation:**	Reviewer 2 / Évaluateur 2
**Name of Applicant / Nom du chercheur:**	Gelinas, Céline
**Application No. / Numéro de demande:**	427389
**Agency / Agence:**	CIHR/IRSC
**Competition / Concours:**	Project Grant/Subvention Projet
**Committee / Comité:**	Psychosocial, Sociocultural & Behavioural Determinants of Health/Déterminants psychosociaux, socioculturels et comportementaux de la santé
**Title / Titre:**	Validation of a new multi-parameter technology to better diagnose pain in the adult intensive care population: The Nociception Level (NOL) Index

Summary of Application/Résumé de la demande:

This project addresses the complex and important issue of assessing pain in adult intensive care units (ICU) in situations where self-report is not an option and mechanical ventilation and/or sedation further complicate assessment.

It will test a newly developed Nociception Level (NOL) index obtained via the Pain Monitoring Device-200 (PMD-200), that was recently approved as medical device in Canada. The device uses a finger probe and disposable sensor which includes four small sensors: a) accelerometer, b) photoplethysmograph, c) galvanic skin response, and d) peripheral temperature. The NOL is a numeric value from 0 to 100 obtained via a linear combination of sensor parameters. Previous research by the team has generated clinical cut offs for anesthetized patients. Pilot work demonstrated the feasibility of the methods and relationships between the NOL with AUC>0.75 for detecting self-reported pain intensity and Critical-Care Pain Observation Tool (CPOT) during chest tube removal in surgical ICU patients.

The aims are to examine the NOL's ability to:

a) detect pain in patients able and unable to self-report, and capable of expressing behaviors or not; and

b) discriminate between non-painful and painful procedures.

Methods: A prospective cohort design will be used. Three samples of patients will be enrolled: a) can communicate (able to self-report), b) cannot communicate but can exhibit behaviors, and c) cannot communicate nor exhibit behaviors. Observations of routine clinical procedures that are painful (e.g., chest tube or drain removal, endotracheal suctioning, arterial or intravenous catheter insertion, wound care) or are not painful (i.e., cuff inflation) will be conducted and the NOL continuously monitored during the procedures. Where possible, self-reported pain intensity will be obtained; observed pain will be assessed with the CPOT.

Receiver Operating Characteristic curve analysis will be conducted with the aim of determining the optimal cut-point for clinically significant pain.

**Review Type / Type d'évaluation:**	Reviewer 2 / Évaluateur 2
**Name of Applicant / Nom du chercheur:**	Gelinas, Céline
**Application No. / Numéro de demande:**	427389
**Agency / Agence:**	CIHR/IRSC
**Competition / Concours:**	Project Grant/Subvention Projet
**Committee / Comité:**	Psychosocial, Sociocultural & Behavioural Determinants of Health/Déterminants psychosociaux, socioculturels et comportementaux de la santé
**Title / Titre:**	Validation of a new multi-parameter technology to better diagnose pain in the adult intensive care population: The Nociception Level (NOL) Index

Strengths and Weaknesses/Forces et faiblesses:

STRENGTHS

A) Significance and Impact of Research

The assessment of pain amongst patients for whom self-report is not possible, or not always an option, is a major challenge for research and clinicians. The availability of a device to assess pain is a major breakthrough in the field. Obtaining additional validity data on the Pain Monitoring Device-200 and determining clinical cut-offs for the NOL is a critical next step in this research and can have important applications clinically.

B) Approaches and Methods

Conducting studies in the ICU is extremely challenging. Use of routine procedures in the study is an excellent approach. Pilot data are promising and support the feasibility of the proposed protocol.

C) Expertise, Experience, and Resources

This team is uniquely qualified to conduct the proposed study. They have experience with conducting pain assessments in the ICU and have done previous work with the Pain Monitoring Device.

The PI, Gélinas, is a nurse researcher and Associate Professor in Nursing at McGill. She has a solid record of grant funding, and an excellent publication record most of her publications related to the topics being investigated in the proposed study. The rest of the team includes other nurses, and physician researchers and knowledge users; nursing students are also involved in the project. The team includes individuals working the ICU who can facilitate access to the patient population.

Overall, the team has the expertise to conducted the proposed study.

WEAKNESSES

A) Significance and Impact of Research

No substantive concerns.

B) Approaches and Methods

The power/sample size computations were a bit confusing. The power calculations are based on running ROC analyses. Later they state a minimal sample size of 43 patients is required for t-tests. This should have been re-worded to state that they have more than sufficient power for these secondary analyses.

Analyses for Group C (unable to provide self-report) focuses just on comparisons between the nociceptive and nonnociceptive procedures. The team should consider using the CPOT data as well.

**Review Type / Type d'évaluation:**	Reviewer 2 / Évaluateur 2
**Name of Applicant / Nom du chercheur:**	Gelinas, Céline
**Application No. / Numéro de demande:**	427389
**Agency / Agence:**	CIHR/IRSC
**Competition / Concours:**	Project Grant/Subvention Projet
**Committee / Comité:**	Psychosocial, Sociocultural & Behavioural Determinants of Health/Déterminants psychosociaux, socioculturels et comportementaux de la santé
**Title / Titre:**	Validation of a new multi-parameter technology to better diagnose pain in the adult intensive care population: The Nociception Level (NOL) Index

Budget Recommendation/Recommandation budgétaire:

It appears reasonable.

Please indicate your appraisal of the integration of sex as a biological variable as a strength, weakness, or not applicable to the proposal./Prière de sélectionner une option pour donner votre évaluation de l'intégration du sexe comme variable biologique en tant que point fort ou point faible de la proposition, ou en tant qu'élément non applicable à la proposition.

- ◉ **Strength/Point fort**
- ○ **Weakness/Point faible**
- ○ **Not applicable/Non applicable**

Please indicate your appraisal of the integration of gender as a socio-cultural determinant of health as a strength, weakness, or not applicable to the proposal./Prière de sélectionner une option pour donner votre évaluation de l'intégration du genre comme déterminant socioculturel de la santé en tant que point fort ou point faible de la proposition, ou en tant qu'élément non applicable à la proposition.

- ◉ **Strength/Point fort**
- ○ **Weakness/Point faible**
- ○ **Not applicable/Non applicable**

**Review Type / Type d'évaluation:**	Reviewer 2 / Évaluateur 2
**Name of Applicant / Nom du chercheur:**	Gelinas, Céline
**Application No. / Numéro de demande:**	427389
**Agency / Agence:**	CIHR/IRSC
**Competition / Concours:**	Project Grant/Subvention Projet
**Committee / Comité:**	Psychosocial, Sociocultural & Behavioural Determinants of Health/Déterminants psychosociaux, socioculturels et comportementaux de la santé
**Title / Titre:**	Validation of a new multi-parameter technology to better diagnose pain in the adult intensive care population: The Nociception Level (NOL) Index

Sex and/or Gender Considerations/Notions de sexe et/ou de genre:

No concerns.

**Review Type / Type d'évaluation:**	Reviewer 3 / Évaluateur 3
**Name of Applicant / Nom du chercheur:**	Gelinas, Céline
**Application No. / Numéro de demande:**	427389
**Agency / Agence:**	CIHR/IRSC
**Competition / Concours:**	Project Grant/Subvention Projet
**Committee / Comité:**	Psychosocial, Sociocultural & Behavioural Determinants of Health/Déterminants psychosociaux, socioculturels et comportementaux de la santé
**Title / Titre:**	Validation of a new multi-parameter technology to better diagnose pain in the adult intensive care population: The Nociception Level (NOL) Index

Adjudication Criteria/Critères de sélection

Significance and Impact of the Research/Importance et impact de la recherche: 3.8

Approaches and Methods/Approches et méthodes: 3.8

Expertise, Experience and Resources/Expertise, expérience et ressources: 4.2

Top/Bottom Selection/Groupe supérieur/inférieur

- ◉ **Top/Groupe supérieur**
- ○ **Bottom/Groupe inférieur**

**Review Type / Type d'évaluation:**	Reviewer 3 / Évaluateur 3
**Name of Applicant / Nom du chercheur:**	Gelinas, Céline
**Application No. / Numéro de demande:**	427389
**Agency / Agence:**	CIHR/IRSC
**Competition / Concours:**	Project Grant/Subvention Projet
**Committee / Comité:**	Psychosocial, Sociocultural & Behavioural Determinants of Health/Déterminants psychosociaux, socioculturels et comportementaux de la santé
**Title / Titre:**	Validation of a new multi-parameter technology to better diagnose pain in the adult intensive care population: The Nociception Level (NOL) Index

Summary of Application/Résumé de la demande:

This study concerns a method for assessing pain in adult intensive care units (ICU) a newly developed Nociception Level (NOL) index, a numeric value calculated from heart rate, heart rate variability, photoplethysmography pulse wave amplitude, skin conductance level, number of skin conductance fluctuations, skin temperature, and their time derivatives. Specific objectives in this study, based on pilot work, are to examine the NOL's ability to detect pain in patients able and unable to self-report, and discriminate between non-painful and painful procedures.

The study will use a prospective cohort design; three samples of patients representative of the adult ICU population who can communicate verbally, cannot communicate but can exhibit behaviors, and can neither communicate nor exhibit behaviors will be recruited. Participants will be observed before, during and 15 minutes after non-painful and painful procedures The expected outcomes include the validation of the NOL in a new context of care which could be used

as an alternative measure of pain in an ICT population.

**Review Type / Type d'évaluation:**	Reviewer 3 / Évaluateur 3
**Name of Applicant / Nom du chercheur:**	Gelinas, Céline
**Application No. / Numéro de demande:**	427389
**Agency / Agence:**	CIHR/IRSC
**Competition / Concours:**	Project Grant/Subvention Projet
**Committee / Comité:**	Psychosocial, Sociocultural & Behavioural Determinants of Health/Déterminants psychosociaux, socioculturels et comportementaux de la santé
**Title / Titre:**	Validation of a new multi-parameter technology to better diagnose pain in the adult intensive care population: The Nociception Level (NOL) Index

Strengths and Weaknesses/Forces et faiblesses:

Strengths

Without doubt this is an important question in a vulnerable population.

The work is also programmatic and led by a strong team with a significant track record in a highly sub-specialized area that is also filled with delicate ethical challenges (e.g. consent from unconscious patients). This has been well thought through.

The team involved here seems well equipped to do this work; laudatory because of the complexity, and one wishes that pain assessment was indeed a priority in this setting. worthy.

Weaknesses

Along the lines of ethics, consent, I did not find mention of what might happen if a patient, after enrollment, becomes conscious and wishes to withdraw. Will that be facilitated if so wished?

For the group C, no-communication and no-behaviour, I was uncertain how the NOL can truly be validated since there is no "gold standard" comparator possible in that group (i.e., no self report, no CPOT). This is more than minor, because for the other two groups we do have ways of assessing pain, and the group where it's most needed C is exactly where it will be hard to determine whether the NOL is doing its job.

As previous reviewers have expressed, not clear to me how feasible recruitment is- especially given exclusion criteria, and the clinical realities of these two ICU units, and complexities of the procedures involved for staff.

**Review Type / Type d'évaluation:**	Reviewer 3 / Évaluateur 3
**Name of Applicant / Nom du chercheur:**	Gelinas, Céline
**Application No. / Numéro de demande:**	427389
**Agency / Agence:**	CIHR/IRSC
**Competition / Concours:**	Project Grant/Subvention Projet
**Committee / Comité:**	Psychosocial, Sociocultural & Behavioural Determinants of Health/Déterminants psychosociaux, socioculturels et comportementaux de la santé
**Title / Titre:**	Validation of a new multi-parameter technology to better diagnose pain in the adult intensive care population: The Nociception Level (NOL) Index

Budget Recommendation/Recommandation budgétaire:

Accepted as described

**Review Type / Type d'évaluation:**	Reviewer 3 / Évaluateur 3
**Name of Applicant / Nom du chercheur:**	Gelinas, Céline
**Application No. / Numéro de demande:**	427389
**Agency / Agence:**	CIHR/IRSC
**Competition / Concours:**	Project Grant/Subvention Projet
**Committee / Comité:**	Psychosocial, Sociocultural & Behavioural Determinants of Health/Déterminants psychosociaux, socioculturels et comportementaux de la santé
**Title / Titre:**	Validation of a new multi-parameter technology to better diagnose pain in the adult intensive care population: The Nociception Level (NOL) Index

Please indicate your appraisal of the integration of sex as a biological variable as a strength, weakness, or not applicable to the proposal./Prière de sélectionner une option pour donner votre évaluation de l'intégration du sexe comme variable biologique en tant que point fort ou point faible de la proposition, ou en tant qu'élément non applicable à la proposition.

- (●) **Strength/Point fort**
- ( ) **Weakness/Point faible**
- ( ) **Not applicable/Non applicable**

Please indicate your appraisal of the integration of gender as a socio-cultural determinant of health as a strength, weakness, or not applicable to the proposal./Prière de sélectionner une option pour donner votre évaluation de l'intégration du genre comme déterminant socioculturel de la santé en tant que point fort ou point faible de la proposition, ou en tant qu'élément non applicable à la proposition.

- (●) **Strength/Point fort**
- ( ) **Weakness/Point faible**
- ( ) **Not applicable/Non applicable**

**Review Type / Type d'évaluation:**	Reviewer 3 / Évaluateur 3
**Name of Applicant / Nom du chercheur:**	Gelinas, Céline
**Application No. / Numéro de demande:**	427389
**Agency / Agence:**	CIHR/IRSC
**Competition / Concours:**	Project Grant/Subvention Projet
**Committee / Comité:**	Psychosocial, Sociocultural & Behavioural Determinants of Health/Déterminants psychosociaux, socioculturels et comportementaux de la santé
**Title / Titre:**	Validation of a new multi-parameter technology to better diagnose pain in the adult intensive care population: The Nociception Level (NOL) Index

Sex and/or Gender Considerations/Notions de sexe et/ou de genre:

The researchers state rightly that sex and gender are important variables to consider in relation to pain. And indeed they do plan to explore the influence of sex and gender on the NOL by calculating descriptive statistics stratified by sex and gender to characterize the study samples, and outcomes, as well as using sex and gender as covariates.

Review Type/Type d'évaluation: SO Notes /Notes de l'agent scientifique
Name of Applicant/Nom du chercheur: Gelinas, Céline
Application No./Numéro de demande: 427389
Agency/Agence: CIHR/IRSC
Competition/Concours: 2019-09-11 Project Grant/Subvention Projet
Committee/Comité: Psychosocial, Sociocultural & Behavioural Determinants of Health/Déterminants psychosociaux, socioculturels et comportementaux de la santé
Title/Titre: Validation of a new multi-parameter technology to better diagnose pain in the adult intensive care population: The Nociception Level (NOL) Index

---

Assessment/Évaluation:

Strengths:

This second resubmission proposes to validate a new measure derived from multiple recordings of physiological parameters – the NOL – for patients in the ICU who cannot verbally communicate their pain experience. The proposal retains all the strengths of the original submission: A highly relevant topic, excellent pilot data, and a study design that will produce clinically relevant findings for a group of critically ill individuals, and eventually other under-served patient categories. The technology is impressive and approved by Health Canada. The power analysis was improved, taking into account differences between patients groups as well as sex and gender considerations. There are strong collaborations with ICUs in the Montreal area and the strong team is well poised to take on the challenges inherent to this ambitious proposal.

Weaknesses:

Feasibility remains of some concern, specifically in terms of obtaining consent from family members, although this was considered to be minor. A discussion of other pain assessment tools would have strengthened the proposal. Implementation of this novel pain measure was discussed among committee members, who suggested paying specific attention to uptake and dissemination.

Budget: No issue.
Term: No issue

**Review Type/Type d'évaluation:**	SO Notes /Notes de l'agent scientifique
**Name of Applicant/Nom du chercheur:**	Gelinas, Céline
**Application No./Numéro de demande:**	427389
**Agency/Agence:**	CIHR/IRSC
**Competition/Concours:**	2019-09-11 Project Grant/Subvention Projet
**Committee/Comité:**	Psychosocial, Sociocultural & Behavioural Determinants of Health/Déterminants psychosociaux, socioculturels et comportementaux de la santé
**Title/Titre:**	Validation of a new multi-parameter technology to better diagnose pain in the adult intensive care population: The Nociception Level (NOL) Index

Assessment/Évaluation:

Eligibility: No issue
Ethics: No issue
